# Adapting a Dehazing System to Haze Conditions by Piece-Wisely Linearizing a Depth Estimator

**DOI:** 10.3390/s22051957

**Published:** 2022-03-02

**Authors:** Dat Ngo, Seungmin Lee, Ui-Jean Kang, Tri Minh Ngo, Gi-Dong Lee, Bongsoon Kang

**Affiliations:** 1 Department of Electronics Engineering, Dong-A University, Busan 49315, Korea; datngo@donga.ac.kr (D.N.); 1672885@donga.ac.kr (S.L.); 2171003@donga.ac.kr (U.-J.K.); gdlee@dau.ac.kr (G.-D.L.); 2 Faculty of Electronics and Telecommunication Engineering, The University of Danang—University of Science and Technology, Danang 550000, Vietnam

**Keywords:** image dehazing, haze condition, piece-wise linearization, hardware implementation

## Abstract

Haze is the most frequently encountered weather condition on the road, and it accounts for a considerable number of car crashes occurring every year. Accordingly, image dehazing has garnered strong interest in recent decades. However, although various algorithms have been developed, a robust dehazing method that can operate reliably in different haze conditions is still in great demand. Therefore, this paper presents a method to adapt a dehazing system to various haze conditions. Under this approach, the proposed method discriminates haze conditions based on the haze density estimate. The discrimination result is then leveraged to form a piece-wise linear weight to modify the depth estimator. Consequently, the proposed method can effectively handle arbitrary input images regardless of their haze condition. This paper also presents a corresponding real-time hardware implementation to facilitate the integration into existing embedded systems. Finally, a comparative assessment against benchmark designs demonstrates the efficacy of the proposed dehazing method and its hardware counterpart.

## 1. Introduction

High-level object recognition systems, which operate in outdoor environments, are subject to weather conditions. At present, especially in Asia, where air pollution poses an increasingly serious problem, haze has dominated other ones, such as rain and snow. As a result, it has rapidly aroused interest from academia and industry. Because the most noticeable haze-induced problem is poor visibility, machine vision systems operating in hazy weather suffer from a sharp decrease in performance. According to a comprehensive investigation of Pei et al. [1], performance reduces proportionally to the haze thickness; thus, rendering image dehazing essential. In this context, image dehazing should be located near the camera to pre-process the image data to improve visibility. Consequently, other high-level algorithms can gain a performance boost as they now operate on the pre-processed data where the haze-induced problem has been alleviated significantly. Nevertheless, image dehazing as a pre-processing step imposes tight constraints on the execution time and algorithmic complexity, leading to the urgent demand for a fast and efficient image dehazing method.

Image dehazing is seemingly in its mature stage, and the focus of recent research is on balancing the trade-off between the algorithmic complexity and the processing time. For example, deep dehazing models often yield state-of-the-art performance at expensive computational and manufacturing costs. This limitation hinders the practical application in real-world embedded systems, where the aforementioned factors are critical. The FAMED-Net developed by Zhang and Tao [2] is a prime example. This network could handle 620×460 images at 35.00 frames per second (fps) on a workstation with Nvidia Titan XP graphics processing units (GPUs). Nevertheless, because the GPU is a high-power-consuming platform that incurs an expensive manufacturing cost, it is inappropriate for real-world embedded systems.

Field-programmable gate arrays (FPGAs) are an alternative supporting real-time processing while not incurring a high-power burden. Compared to GPUs, FPGAs are the preferable choice for facilitating the integration of image processing techniques into embedded systems, as demonstrated by many studies in the literature [3,4,5,6]. The algorithm can be tailored to meet the power and timing budgets due to the reconfigurability; hence, a fast and energy-efficient implementation. The main disadvantage of employing FPGAs is the burdensome design methodology; however, it is outweighed by the aforementioned advantage.

This study aims to develop a robust and efficient algorithm exhibiting satisfactory performance while retaining real-time processing capability. The proposed algorithm is grounded on the color attenuation prior (CAP), discovered by Zhu et al. [7], to establish a linear depth estimator, which can provide a fast and reliable depth estimate from the image saturation and brightness. Furthermore, the proposed algorithm leverages the haziness degree evaluator [8] to discriminate the haze condition of the input image. The discrimination result then serves as a basis for piece-wisely linearizing the depth estimator, equipping the proposed algorithm with an ability to remove haze in a haze-condition-appropriate manner. Finally, this paper presents a corresponding FPGA implementation to facilitate the application in real-world embedded systems. The main contributions are twofold:

- an elegant solution to form a piece-wise linear weight from the haze density estimate to improve the robustness of a dehazing algorithm, and
- a real-time high-performance hardware accelerator that can handle DCI 4K images at 30.94 fps.

The remaining of this paper is as follows. The next section investigates related work that removes haze from a single input image. Section 3 provides a detailed description of the proposed algorithm, while Section 4 presents a comparative evaluation to demonstrate its efficacy. After that, Section 5 briefly introduces the hardware implementation methodology and then delves deeply into the hardware architecture of the proposed algorithm. This section also presents the hardware implementation results to verify the real-time processing. Finally, Section 6 concludes the paper.

## 2. Related Work

The vast majority of image dehazing algorithms hitherto developed are grounded on the simplified Koschmieder model [9], which describes the hazy image formation in the atmosphere as follows:

(1) $$I\left(x\right)=J\left(x\right)t\left(x\right)+A\left[1-t(x)\right],$$

where *x* denotes the spatial coordinates of image pixels, I the input image, J the scene radiance, A the global atmospheric light, and *t* the transmission map (or, equivalently, transmittance). The boldfaced representations of I, J, and A signify that these variables have three color channels, corresponding to three typical image sensors sensitive to red, green, and blue wavelengths. Meanwhile, the plain representation of the transmittance signifies that it is a single channel variable inversely proportional to the haze concentration. This depth-dependent variable is expressed as below:

(2) $$t\left(x\right)=exp\left[-\beta_{sc}d\left(x\right)\right],$$

where $\beta_{sc}$ is the atmospheric scattering coefficient, and *d* is the scene depth. Back to Equation (1), the first term on the right-hand side, J(x)t(x), represents the attenuation of wavelengths reflected from the object’s surface, widely referred to as the *direct attenuation*. The remaining term, $A\left[1-t(x)\right]$, represents the amount of light scattered directly into the image sensors, referred to as the *airlight*.

The objective of image dehazing is to estimate two unknowns, A and *t*, from a single input I for recovering the scene radiance J. In one of the pioneering attempts, He et al. [10] fulfilled this objective through the dark channel prior (DCP), which states that non-sky image patches tend to possess an extremely dark channel whose intensities approximate zero. Based on the DCP, they estimated the transmittance by assuming that the dark channel of the scene radiance was zero and that the transmittance was locally smooth. These assumptions yielded an accurate estimate that was unfortunately affected by block artifacts; hence, they employed computationally intensive soft-matting [11] for refinement. They also developed the guided filtering technique [12] two years later as an alternative to soft-matting to lower algorithmic complexity. Furthermore, He et al. [10] presented a robust approach to estimate atmospheric light. Under this approach, the top 0.1% brightest pixels from the dark channel are first selected as a search domain, from which the pixel with the highest intensity in the red-green-blue (RGB) color space is singled out as the atmospheric light. The dehazing method developed by He et al. [10] is well-recognized for its excellent performance in general. However, it may cause color distortion for images with a broad sky, where the DCP does not hold.

In another attempt, Zhu et al. [7] discovered the CAP, which stated that the scene depth strongly correlated with the difference between saturation and brightness. After that, they modeled this correlation by a linear equation, wherein unknown coefficients were estimated using maximum likelihood estimates (MLE). Meanwhile, Zhu et al. [7] adopted an approach similar to that of He et al. [10] to predict the atmospheric light, except they employed the scene depth instead of the dark channel. This CAP-based method is fast and efficient; however, Ngo et al. [13] investigated this method thoroughly and discovered several limitations, such as background noise and color distortion. A year later, Ngo et al. [14] further improved the CAP-based method by adding a new feature: atmospheric light compensation for remedying the post-dehazing false enlargement of bright objects.

Moreover, researchers also leveraged other haze-relevant features to estimate the transmittance. Tang et al. [15] employed the random forest regression technique to infer the transmittance from a set of multiscale features, including the dark channel, hue disparity, contrast, and saturation. Similarly, Ngo et al. [16] utilized the Nelder-Mead direct search algorithm to seek a transmittance estimate that maximized the product of contrast energy, image entropy, and sharpness. Although these two methods could deliver satisfactory results, they significantly prolonged the execution time, limiting their breadth of application.

As image dehazing from a single image is an ill-posed and challenging problem, researchers have attempted to exploit the powerful representation capability of deep neural networks. Cai et al. [17] designed a simple three-layer convolutional neural network (CNN) that sequentially performed the following three operations: feature extraction, multiscale mapping, and nonlinear regression to predict the transmittance. These three operations form the fundamental basis of deep-learning-based dehazing, and subsequent studies have mainly improved that basis for attaining better performance. For example, Ren et al. [18] focused on the multiscale mapping operation, in which they utilized two CNNs: a coarse-scale CNN with large kernel sizes and a fine-scale CNN with small kernel sizes. The experimental results demonstrated that this coarse-to-fine multiscale mapping significantly boosted performance. Nevertheless, these deep CNNs are prone to the domain shift problem because they utilize synthetic data for training. Another limitation is their inherent heavy computational burden, which requires high-power-consuming GPUs for training and inference.

**Figure 1:**
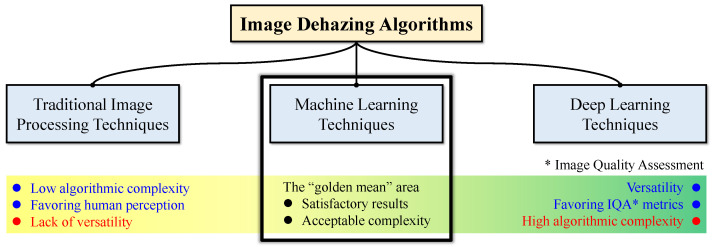
Image dehazing and its corresponding three main categories divided based on the core technique.

In conclusion, image dehazing algorithms generally fall into three main categories: traditional image processing, machine learning, and deep learning. In the DCP-based methods, He et al. [10,12] recovered the scene radiance by hand-engineered techniques. They did not utilize sample data to learn any implicit or explicit correlation between hazy and haze-free images. Conversely, in the CAP-based methods, Zhu et al. [7] and Ngo et al. [13,14] collected sample data and employed MLE to learn the coefficients for transmittance estimation. As a result, the DCP-based methods belong to the first category, and the CAP-based methods fall into the second. Meanwhile, the methods proposed by Cai et al. [17] and Ren et al. [18] are in the third category as they depend on deep CNNs. The main difference between machine-learning-based and deep-learning-based methods is that the latter leverages the powerful feature-extracting capability of CNNs, while the former still employs hand-crafted features. Figure 1 illustrates these three categories with their corresponding advantages and disadvantages. It can be observed that the second category is seemingly the “golden mean,” which exhibits satisfactory results while not incurring a heavy computational burden. Therefore, the proposed algorithm only employs traditional image processing and machine learning techniques to balance the execution time and the restoration quality. Interested readers are referred to a systematic review in [19] for an in-depth body of knowledge.

## 3. Proposed Algorithm

**Figure 2:**
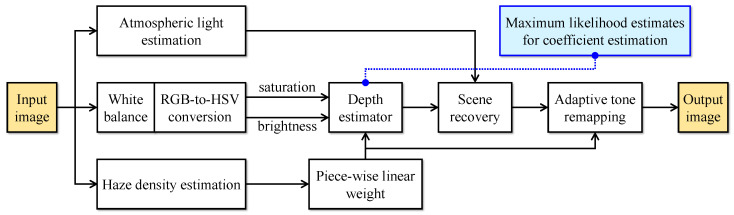
Block diagram of the proposed image dehazing algorithm. RGB stands for red-green-blue, and HSV hue-saturation-value.

The proposed image dehazing algorithm is grounded on the CAP, which forms a basis of scene depth estimation using saturation and brightness. The novelty lies in using the haze density estimate to linearize the depth estimator piece-wisely to achieve robustness against different haze conditions. Figure 2 depicts the block diagram of the proposed algorithm. It is worth noting that the MLE for estimating the coefficients of the depth estimator does not affect the algorithmic complexity because it is performed separately from the main computation. In general, the proposed algorithm accepts an arbitrary input image and performs the following three operations concurrently:

- atmospheric light estimation using the approximated version of the quad-tree decomposition [20],
- white balance, immediately followed by saturation and brightness extraction, and
- haze density estimation using the haziness degree evaluator [8].

After that, the saturation and brightness serve as inputs to the depth estimator, whose coefficients are the results of applying MLE to sample data. At the same time, the haze density estimate serves as a basis for haze condition discrimination, which classifies the input image as haze-free, mildly hazy, moderately hazy, or densely hazy. Then, based on the discrimination result, a corresponding weight factor modifies the scene depth estimate to control the scene recovery implicitly. More specifically, the recovered scene radiance is the input image if it is haze-free. Otherwise, the weight factor proportionally modifies the dehazing power to ensure that the haze is removed appropriately to the haze condition. For example, if the input image is affected by mild haze, the weight factor reduces the dehazing power lest excess-haze-removal-induced artifacts arise. Finally, the adaptive tone remapping (ATR) method [21] post-processes the recovered scene radiance to compensate for the probable dynamic range reduction. Notably, the weight factor also modifies the ATR to guarantee that the dynamic range expansion degree is appropriate to the amount of haze that has been removed.

### 3.1. Scene Depth Estimation

Although the scene depth estimation is fundamentally similar to that of the original CAP-based method [7], this study incorporates two following modifications:

- employing the enhanced equidistribution [14] instead of the standard uniform distribution to create the synthetic sample data, and
- adopting the mini-batch gradient ascent algorithm with an adaptive learning rate to achieve a fast convergence rate.

First of all, the depth estimator developed by Zhu et al. [7] is given below:

(3) $$d\left(x\right)=\theta_{0}+\theta_{1}s\left(x\right)+\theta_{2}v\left(x\right)+\varepsilon \left(x\right),$$

where *s* denotes saturation, *v* brightness, $\left\{\theta_{0},\theta_{1},\theta_{2}\right\}$ coefficients, and ε inherent error of the model. Zhu et al. [7] assumed that ε followed Gaussian distribution with zero mean and $\sigma^{2}$ variance. Mathematically, it can be expressed as $\varepsilon ∼N(0,\sigma^{2})$. Thus, it can be deduced from Equation (3) that $d∼N(\theta_{0}+\theta_{1}s+\theta_{2}v,\sigma^{2})$. Zhu et al. [7] further assumed that the error associated with each pixel was independent and identically distributed. Consequently, they derived the following formulas for calculating the likelihood function *L* and coefficients:

(4) $$\begin{array}{ccc} L & = & \prod_{i=1}^{N}\frac{1}{\sigma \sqrt{2\pi }}exp\left\{-\frac{{\left[d\left(i\right)-\theta_{0}-\theta_{1}s\left(i\right)-\theta_{2}v\left(i\right)\right]}^{2}}{2\sigma^{2}}\right\}, \end{array}$$

(5) $$\begin{array}{ccc} \theta_{0} & ≔ & \theta_{0}+\rho \frac{\partial ln(L)}{\partial \theta_{0}}, \end{array}$$

(6) $$\begin{array}{ccc} \theta_{1} & ≔ & \theta_{1}+\rho \frac{\partial ln(L)}{\partial \theta_{1}}, \end{array}$$

(7) $$\begin{array}{ccc} \theta_{2} & ≔ & \theta_{2}+\rho \frac{\partial ln(L)}{\partial \theta_{2}}, \end{array}$$

where *N* denotes the total number of pixels in the sample data, ρ the learning rate, and ln(·) the natural logarithm. Notably, the symbol ≔ is analogous to the word “become,” which signifies that the coefficients are updated dynamically.

**Figure 3:**
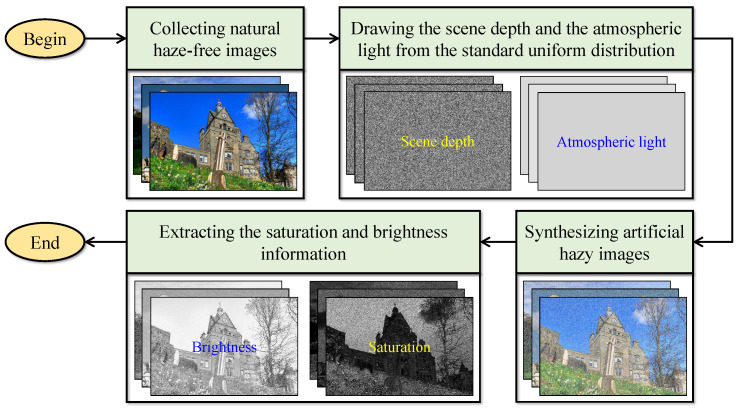
Procedure for synthesizing artificial sample data to learn the model’s coefficients in the method of Zhu et al. [7].

Based on the above description, the sample data consisting of saturation, brightness, and the reference scene depth is essential to learn the coefficients. However, in practice, the commercial depth cameras cannot capture the scene depth reliably, giving rise to the sheer impracticality of collecting such required sample data. Zhu et al. [7] then resorted to utilizing the synthetic data for coefficient estimation. They first collected natural haze-free images from the Internet. Then, they drew the scene depth and the atmospheric light from the standard uniform distribution. By substituting these two variables into the simplified Koschmieder model, they synthesized the artificial hazy images, from which they derived the saturation and brightness information. Figure 3 visualizes this procedure for ease of understanding.

**Figure 4:**
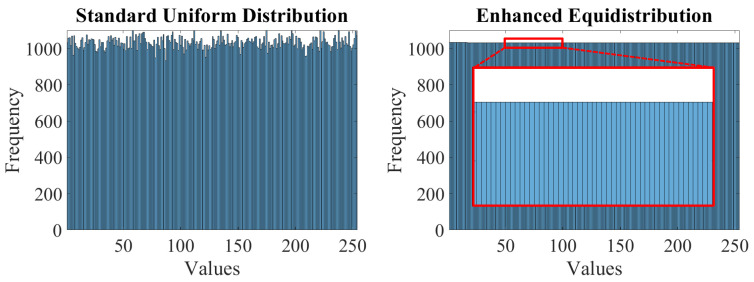
Histograms of random data drawn from the standard uniform distribution and the enhanced equidistribution. A 512 × 512 scene depth with 262,144 data points was assumed.

A minor flaw in the procedure described above is that random number generators do not guarantee a true uniform distribution. Thus, this study utilizes the enhanced equidistribution [14], which ensures that the random numbers are uniformly distributed. Figure 4 illustrates the histograms of 262,144 random data points, corresponding to a 512×512 scene depth, drawn from the standard uniform distribution and the enhanced equidistribution. It is conspicuous that the enhanced equidistribution strictly enforces the uniform distribution, yielding a variance much smaller than that of the standard uniform distribution. Hence, the procedure for synthesizing artificial sample data in this study is almost similar to the one illustrated in Figure 3, except that the enhanced equidistribution replaces the standard uniform distribution.

Given the sample data, Zhu et al. [7] utilized the gradient ascent algorithm to locate the maximum log-likelihood value, which yielded the coefficient estimates. In the follow-up study, Ngo et al. [14] employed the mini-batch gradient ascent algorithm, which was more robust than its naive counterpart utilized by Zhu et al. [7]. However, because authors adopted a constant learning rate in these two methods, they resorted to adjusting this hyper-parameter repeatedly to find the plateau of the log-likelihood function. As this process is time-consuming, this study presents a simple method for updating the learning rate dynamically to achieve a fast convergence rate.

**Figure 5:**
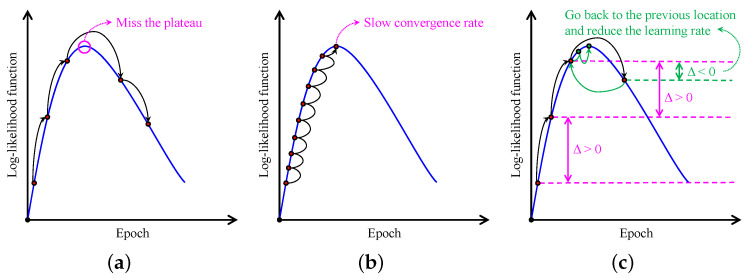
Illustration of the gradient ascent algorithm with (**a**) the large learning rate, (**b**) small learning rate, and (**c**) proposed adaptive learning rate.

Figure 5 illustrates the impact of the learning rate on the convergence of the gradient ascent algorithm. As depicted in Figure 5a, using a large learning rate leads to drastic updates of the log-likelihood function, possibly missing the plateau or even causing outright divergence. In contrast, adopting a small learning rate ensures convergence, but it is highly time-consuming, as illustrated in Figure 5b. Therefore, this study leverages the difference between two successive log-likelihood values (denoted as Δ in Figure 5c) to update the learning rate. More specifically, if Δ is positive, the learning rate remains the same in the next epoch. Otherwise, the training restores to the previous location, and reduces the learning rate by a factor of ξ (ξ=2 in this study). Thus, the proposed learning-rate-updating scheme guarantees that the log-likelihood eventually reaches the plateau, that is, when Δ falls beneath a pre-determined threshold (or ideally becomes zero). Consequently, it is convenient to initialize the learning rate with an arbitrarily large value to shorten the training time.

### 3.2. Piece-Wise Linear Weight

**Figure 6:**
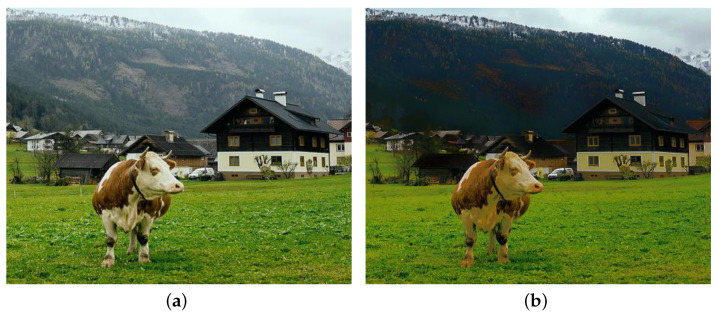
Illustration of declining depth perception when applying image dehazing on a haze-free image. (**a**) Outdoor haze-free image and (**b**) its corresponding dehazing result yielded by the algorithm of Zhu et al. [7].

The scene depth is an inherent characteristic of natural outdoor images regardless of whether they are affected by haze or not. In addition, Equation (2) demonstrates that the haze concentration increases along with the scene depth. Hence, it can be deduced that haze is an indispensable part of those images, and its presence gives the observers the feeling of depth. He et al. [10] mentioned this interesting phenomenon in their work, and referred to it as *aerial perspective*. Although haze exists in the so-called haze-free images, performing image dehazing, in this case, declines the depth perception, as illustrated in Figure 6. The distant haze in Figure 6a is a fundamental cue for observers to perceive that the house is closer to the camera than the mountain. In Figure 6b, this beneficial haze layer has disappeared as a result of applying the algorithm of Zhu et al. [7]; thus, posing considerable difficulties for observers to distinguish how far away the house and the mountain are. In this example of an outdoor landscape image, the problem of declining depth perception is of little importance. However, if this problem arises in driver-assisting systems, serious consequences may occur.

This study aims to remedy the above-mentioned problem by modifying the scene depth estimate according to the haze condition. More precisely, this study leverages the haziness degree evaluator (HDE) [8] to quantify the haze concentration quickly and accurately through a closed-form formula. Details of the HDE calculation can be found in Appendix A. Then, based on the HDE’s result, this study forms a piece-wise linear weight reflecting the haze condition of the input image. After that, this weight factor modifies the scene depth estimate to adjust the dehazing power implicitly. The detailed description is as follows.

The HDE quantifies the haziness degree of an arbitrary image by solving an analytically solvable optimization that maximizes the image’s saturation, brightness, and sharpness while minimizing the dark channel. The result is a closed-form expression of the haze concentration estimate, denoted as $\Gamma_{I}$. This value varies between zero and unity, and is proportional to the haze concentration. Accordingly, this study utilizes a pair of thresholds to partition the value range of $\Gamma_{I}$ into three segments: haze-free, hazy, and densely hazy. In the first segment, the proposed algorithm discriminates the input image as haze-free; hence, it is necessary to skip the dehazing process to avoid declining depth perception. Meanwhile, according to its $\Gamma_{I}$ value, the input image is mildly or moderately hazy in the second segment. Correspondingly, the proposed algorithm needs to control the dehazing power lest post-dehazing artifacts arise due to excess haze removal. Finally, in the third segment, the proposed algorithm regards the input image as densely hazy, and thus unleashes the full dehazing power.

For the above-described purpose, this study formulates the piece-wise linear weight $\omega_{I}$ as below:

(8) $$\omega_{I}=\left\{ \begin{array}{cc} 0 & \Gamma_{I}<\Gamma_{1}\\ \frac{\Gamma_{I}-\Gamma_{1}}{\Gamma_{2}-\Gamma_{1}} & \Gamma_{1}\leq \Gamma_{I}\leq \Gamma_{2}\\ 1 & \Gamma_{I}>\Gamma_{2} \end{array}\right.,$$

where $\left\{\Gamma_{1},\Gamma_{2}\right\}$ denotes a pair of thresholds for segment partition. The weight factor $\omega_{I}$ is then multiplied directly by the scene depth estimate *d* in Equation (3). As a result, if $\Gamma_{I}<\Gamma_{1}$, the proposed algorithm classifies the input image as haze-free and yields $\omega_{I}=0$. This value forces the output of the depth estimator to be zero, that is, d(x)=0, ∀x∈Ψ, with Ψ being the entire image domain. Accordingly, Equation (2) yields t(x)=1, ∀x∈Ψ. Substituting this transmittance value into Equation (1) cancels out the airlight, resulting in I(x)=J(x), ∀x∈Ψ. Hence, the piece-wise linear weight guarantees that haze-free images do not undergo any modifications. This desirable behavior renders the proposed algorithm robust against the problem of declining depth perception.

If $\Gamma_{1}\leq \Gamma_{I}\leq \Gamma_{2}$, the input image is discriminated as hazy, and its corresponding haze condition would be either mildly or moderately hazy depending on the $\Gamma_{I}$ value. In this case, the weight factor varies between zero and unity, and it implicitly modifies the dehazing power of the proposed algorithm. The higher the $\omega_{I}$ is, the greater degree to which image dehazing is performed. Finally, if $\Gamma_{I}>\Gamma_{2}$, the proposed algorithm discriminates the input image as densely hazy, and it sets the weight factor to unity to unleash the full dehazing power.

**Figure 7:**
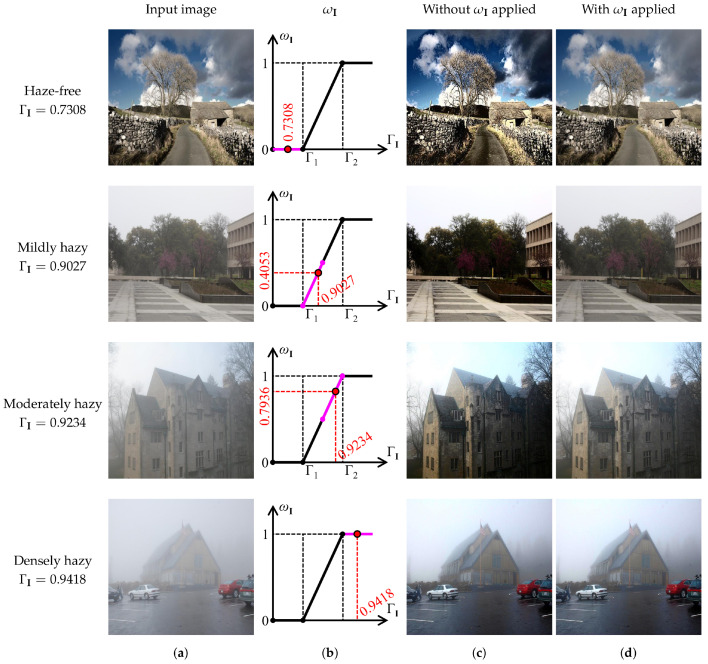
Illustration of the beneficial effects of the proposed piece-wise linear weight. (**a**) Arbitrary input images and their corresponding (**b**) weight factors, (**c**) dehazing results *without* weight factors applied, and (**d**) dehazing results *with* weight factors applied. The pair of thresholds $\left\{\Gamma_{1},\Gamma_{2}\right\}$ was set to {0.8811,0.9344}.

Figure 7 demonstrates the efficacy of adopting the piece-wise linear weight to adapt the proposed algorithm to different haze conditions. Concerning the haze-free image in the first row, it can be observed that the proposed algorithm has discriminated the haze condition correctly, as witnessed by $\Gamma_{I}=0.7308<\Gamma_{1}=0.8811$. Accordingly, the corresponding weight factor guides the proposed algorithm to leave this image unchanged, avoiding the problem of declining depth perception that arises in Figure 7c.

Similarly, hazy images in the second and third rows are classified as mildly and moderately hazy because their corresponding $\Gamma_{I}$ value lies between $\Gamma_{1}$ and $\Gamma_{2}$. The weight factor then modifies the dehazing power to ensure that dehazing results do not incur any visually unpleasant artifacts. By contrast, results without applying the piece-wise linear weight suffer from post-dehazing artifacts, resulting from the excess haze removal. Witness the loss of fine details near the tree twigs and leaves. Finally, concerning the last row of Figure 7, the proposed algorithm has correctly classified the input image as densely hazy, as verified by $\Gamma_{I}=0.9418>\Gamma_{2}=0.9344$. Therefore, the corresponding weight factor of unity signifies that the input image undergoes a full-scale dehazing, identical to when the piece-wise linear weight is not adopted.

### 3.3. Atmospheric Light Estimation

Outdoor imaging is highly problematic because it depends on environmental and artificial factors, such as illuminating sources, weather conditions, and camera shake. Notably, the interference of artificial illumination may render the atmospheric light estimate incorrect. Ngo et al. [14] investigated this problem and demonstrated that the quad-tree decomposition algorithm [20] was an effective solution. This algorithm decomposes the input image into quarters, and repeats the decomposition on the quarter with the highest average luminance. As bright objects are usually located in high contrast regions, the iterative decomposition effectively alleviates their untoward effect on the estimation accuracy. Consequently, the quad-tree decomposition algorithm is robust against the interference of artificial light sources. However, it incurs a heavy memory burden when implemented on reconfigurable platforms for real-time verification. The main cause is the iterative decomposition requiring many frame buffers. Therefore, Ngo et al. [14] also presented an approximated version not requiring any frame buffers. This study then utilizes the frame-buffer-free version to facilitate the real-time implementation presented later in Section 5.

**Figure 8:**
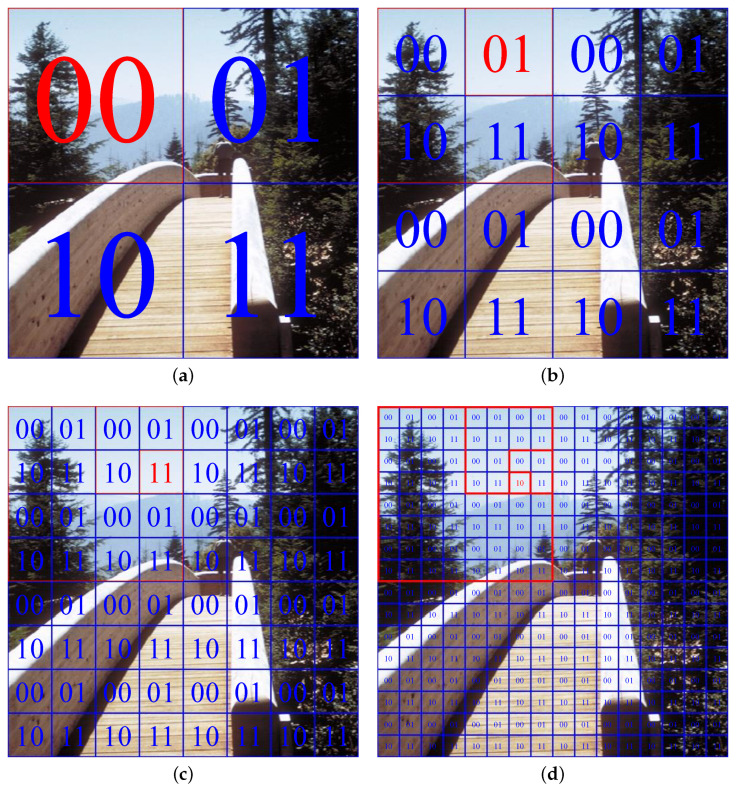
Illustration of the approximated quad-tree decomposition algorithm at (**a**) first, (**b**) second, (**c**) third, and (**d**) fourth levels. The selected quarter is displayed in red.

Ngo et al. [14] approximated the quad-tree decomposition algorithm by fixing the number of times to decompose the image at four. After that, they labeled each group of quarters at four decomposition levels using four 2-bit combinations: 00, 01, 10, and 11, as illustrated in Figure 8. The highest average luminance is still the criterion for decomposition (or, equivalently, selecting one of four combinations). In Figure 8, the red label denotes the selected quarter. Accordingly, this algorithm selects 00, 01, 11, and 10 at four levels in Figure 8a–d, respectively. Then, it combines these 2-bit labels into an 8-bit address that indicates the quarter containing the atmospheric light estimate. More precisely, the most significant bits of four 2-bit labels form the most significant 4-bit part, while the remaining least significant bits form the least significant 4-bit part of the address. In this example, the 8-bit address is 00110110, which indicates that the fifty-fourth quarter away from the top-left (in the left-to-right and top-to-bottom direction) in Figure 8d contains the atmospheric light estimate. Furthermore, in the course of finding 2-bit labels at each level, this algorithm locates all 256 possible candidates for the atmospheric light estimate at the fourth level. It then stores them in random-access memories (RAMs) and utilizes the previous 8-bit address to read the chosen one. As presented thus far, all constituent operations of this approximated version occur concurrently, supporting a greater degree of parallelism than the original quad-tree decomposition. At the same time, these operations do not require any frame buffers.

Concerning the image in Figure 8, it can be observed that the approximated quad-tree decomposition algorithm yields an accurate atmospheric light estimate. However, this estimate possesses smaller intensities than the shiny barrier. Ngo et al. [14] demonstrated that this issue caused the post-dehazing false enlargement of bright objects. As a result, they devised a compensation scheme that scaled up the atmospheric light estimate A to remedy that visually unpleasant problem, as shown in Equation (9) below:

(9) $$\hat{A}=A+\omega_{A}\left\{\max_{\forall x\in \Psi }\left[\max_{c\in \{R,G,B\}}I^{c}\left(x\right)\right]-\max_{c\in \{R,G,B\}}\left(A^{c}\right)\right\},$$

where $\hat{A}$ denotes the compensated atmospheric light estimate, $\omega_{A}$ is the user-defined scaling factor to adjust the compensation amount, and *c* represents the color channel. The $\max_{c\in \{R,G,B\}}\left(\cdot \right)$ operation yields the channel-wise maximum intensity, and the $\max_{\forall x\in \Psi }\left(\cdot \right)$ operation yields the largest intensity over the entire image. Consequently, if the image contains a single illuminating source, the compensation term becomes zero, resulting in $\hat{A}=A$. However, if the image contains multiple illuminating sources, this term is positive, and Equation (9) scales up the atmospheric light estimate accordingly to prevent the post-dehazing false enlargement problem.

### 3.4. Scene Radiance Recovery and Post-Processing

Given the scene depth and atmospheric light estimates, the proposed algorithm employs the following equation to recover the scene radiance:

(10) $$J\left(x\right)=\frac{I\left(x\right)-\hat{A}}{exp\left[-\beta_{sc}\omega_{I}d\left(x\right)\right]}+\hat{A}.$$

**Figure 9:**
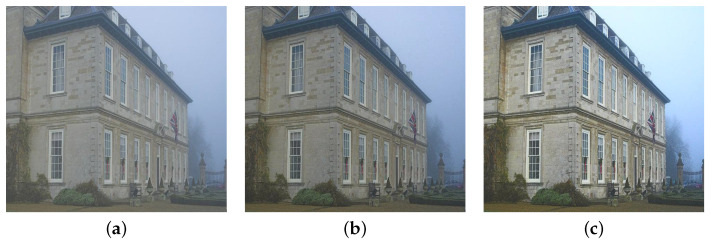
Illustration of tone remapping as an effective solution to dynamic range reduction. (**a**) Hazy image, and its corresponding dehazing results (**b**) *without* and (**c**) *with* tone remapping.

Theoretically, Equation (10) suffices for fulfilling the objective of image dehazing. However, in practice, the dynamic range of the scene radiance reduces significantly due to the inherent quantization error in digital computations, as Figure 9b illustrates. Therefore, this study utilizes the ATR [21] to compensate for that problem. The ATR leverages the cumulative distribution function of the luminance channel to perform the enhancement. It thereafter emphasizes the chrominance information proportionally to ensure that color distortion does not occur. This study also exploits the weight factor $\omega_{I}$ to modify the ATR as follows:

(11) $$\begin{array}{ccc} L_{e}\left(x\right) & = & L\left(x\right)+\omega_{I}G_{L}\left(x\right)W_{L}\left(x\right), \end{array}$$

(12) $$\begin{array}{ccc} C_{e}\left(x\right) & = & C\left(x\right)+G_{C}\left(x\right)W_{C}\left(x\right)+0.5, \end{array}$$

where *L* and *C* denote the luminance and chrominance information of the recovered scene radiance, $L_{e}$ and $C_{e}$ the enhanced luminance and chrominance, $G_{L}$ and $G_{C}$ the luminance and chrominance gains, and $W_{L}$ and $W_{C}$ the adaptive luminance and chrominance weights. Notably, the constant 0.5 in Equation (12) is an offset because the chrominance information must be zero-centered before undergoing the ATR. Details of the calculation of $G_{L}$, $G_{C}$, $W_{L}$, and $W_{C}$ can be found in Appendix B. As $G_{C}=\left(L_{e}/L\right)\cdot C$, chrominance emphasis is proportional to luminance enhancement, and thus the weight factor $\omega_{I}$ adjusts the ATR to the haze condition.

As mentioned in Section 3.2, $\omega_{I}=0$ for haze-free images forces the ATR to perform no enhancement and emphasis, leaving haze-free images intact. This course of action is desirable because post-processing haze-free images is subject to over-saturation. Conversely, $\omega_{I}=1$ for densely hazy images returns the ATR to its original form in [21], which maximally compensates for dynamic range reduction. Lastly, $0<\omega_{I}<1$ modifies the ATR linearly to ensure a haze-condition-appropriate performance for mildly and moderately hazy images. Figure 9 demonstrates that this post-processing step effectively solves the dynamic range reduction.

The dehazing result without ATR applied in Figure 9b appears somber, whereas the result with ATR applied in Figure 9c exhibits the complete opposite. The expansion of the dynamic range is easily noticeable. Witness the vines in the bottom-left corner, the bricks, and the roof.

## 4. Experimental Results

This section compares the proposed algorithm with six benchmark methods whose general description is tabulated in Table 1. It can be observed that this study selects from each image dehazing category two typical algorithms for a comprehensive assessment. Regarding the traditional image processing category, the two benchmark algorithms are developed by Tarel and Hautiere [22] and He et al. [10]. Next, this study selects two CAP-based methods, developed by Zhu et al. [7] and Ngo et al. [14], to typify the machine learning category. Finally, two well-recognized CNNs designed by Cai et al. [17] and Ren et al. [18] are the last two benchmark methods in this assessment.

### 4.1. Parameter Configuration

Before presenting the assessment results, it is worth providing the parameter configuration of the proposed algorithm for reproducibility. As tabulated in Table 2, for the first step of scene depth estimation, the best coefficient values obtained using the mini-batch gradient ascent algorithm with the proposed adaptive learning rate are $\left\{0.1800,1.0147,-0.7350\right\}$. Then, the next step of constructing the piece-wise linear weight is parameterized by a pair of thresholds $\left\{\Gamma_{1},\Gamma_{2}\right\}$, whose values are configured as $\left\{0.8811,0.9344\right\}$. The last parameter is the scaling factor $\omega_{A}$ in the atmospheric compensation scheme, and this study sets it to 0.6000.

Concerning six benchmark algorithms, this study utilizes the default configuration provided by their corresponding authors. The DCP-based algorithm of He et al. [10] can be taken as an example. In the publicized code, authors set the kernel size of spatial filtering operations to 15, the constant representing the aerial perspective phenomenon to 0.9500, and the regularization parameter of soft-matting to 0.0001. This configuration is consistent with the description in [10].

### 4.2. Qualitative Evaluation

**Figure 10:**
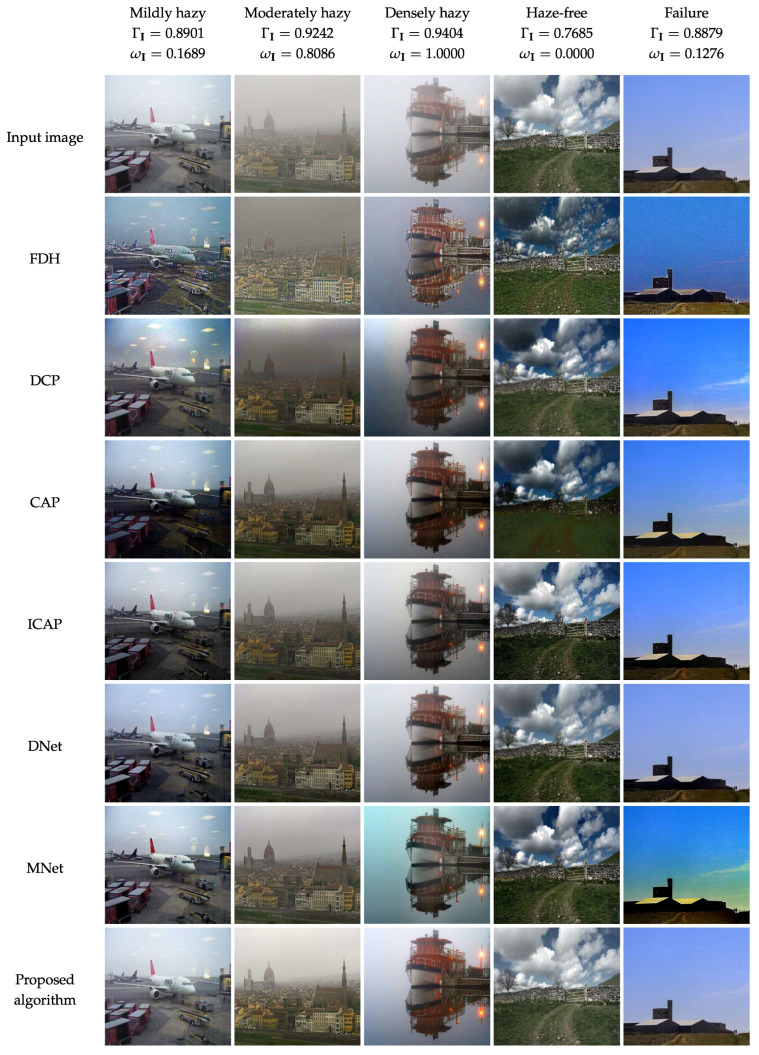
Qualitative comparison of the proposed algorithm with six benchmark methods on images with different haze conditions.

As all six benchmark algorithms generally deliver satisfactory performance, this section only presents the qualitative evaluation results on images that may induce post-dehazing degradation, such as halo artifacts, color distortion, and background noise. Figure 10 illustrates the dehazing results of those algorithms on four different haze conditions: haze-free, mildly hazy, moderately hazy, and densely hazy. In addition, the last column of “failure” represents the case when the proposed algorithm incorrectly discriminates the haze-free input image as hazy. Figure 10 also provides the $\Gamma_{I}$ value corresponding to each haze condition label for ease of confirmation. For example, the proposed algorithm estimates the haze concentration of the haze-free image on the fourth column as $\Gamma_{I}=0.7685$. Because this value is less than the threshold $\Gamma_{1}=0.8811$, it can be confirmed that the discrimination result reflects the true haze condition.

One of the most noticeable problems that many image dehazing algorithms incur is that they usually exhibit too strong dehazing power on mildly hazy images. Accordingly, their dehazing results do not favor human perception. For example, the results of FDH and DCP for mildly hazy images suffer from color distortion in the sky. Concerning the result of CAP, this problem becomes less severe; however, the lower part is too dark due to excess haze removal. Meanwhile, the remaining results of ICAP, DNet, and MNet are more favorable, albeit with slight degradation. Conversely, the proposed algorithm has correctly perceived that the input image is mildly hazy. Thus, it generates a small weight factor [$\omega_{I}=\left(0.8901-0.8811\right)/\left(0.9344-0.8811\right)\approx 0.1689$] to weaken the dehazing power lest any artifacts arise, as illustrated in the last row of Figure 10.

When the input image is moderately hazy, the problem mentioned above appears to be less severe than the case of mildly hazy images. Even though results of FDH and DCP still exhibit some inherent degradation, such as halo artifacts and color distortion, the dehazing performance, in general, is passable. The same interpretation applies to the case when the input image is densely hazy, except for MNet whose result is severely affected by color distortion. Regarding these two cases, the proposed algorithm, with the depth estimator being piece-wisely linearized by the haze condition, produces visually satisfying results without any noticeable post-dehazing artifacts.

The most distinguishable feature of the proposed algorithm compared with benchmark methods is the ability to handle haze-free images without causing any untoward degradation, as demonstrated in the fourth column of Figure 10. This haze-free image contains a thin haze layer that is beneficial for the human visual system to perceive depth information. However, benchmark methods remove it from the input image, causing the problem of declining depth perception. Notably, deep-learning-based methods incur a less severe problem than traditional-image-processing-based and machine-learning-based methods, attributed to the powerful representation capability of CNN. In contrast, the proposed algorithm has correctly discriminated the haze condition, and adjusted the weight factor correspondingly to keep the image unchanged.

Nonetheless, when the input image contains a broad and homogeneous background, the proposed algorithm tends to misclassify its haze condition. In the last column of Figure 10, it can be observed that the proposed algorithm has incorrectly classified a haze-free image as mildly hazy. Accordingly, the result has undergone a dehazing procedure, which might reduce the perceptual visibility. Fortunately, the last column of Figure 10 demonstrates that the proposed algorithm virtually retains the original visibility, because the $\Gamma_{I}$ value of 0.8879 only results in a small weight factor of 0.1276. Meanwhile, color distortion or excess haze removal is easily noticeable in the results of six benchmark methods.

Through this comprehensive assessment, it can be concluded that the proposed algorithm performs better than six benchmark methods because of its ability to handle images with different haze conditions. This beneficial ability directly results from using the haze density estimate to piece-wisely linearize the depth estimator.

### 4.3. Quantitative Evaluation

This section evaluates the proposed algorithm against benchmark methods using image quality assessment (IQA) metrics on publicly available datasets to complete the performance assessment. Firstly, IQA metrics utilized therein are the feature similarity index extended to color images (FSIMc) [23] and the tone-mapped image quality index (TMQI) [24]. The former metric assesses the structural similarity between the dehazing result and its corresponding ground-truth reference. It yields a score between zero and unity, wherein the higher, the better. Meanwhile, the latter metric accounts for the multiscale structural fidelity and statistical naturalness between those two images. It also yields a normalized score, wherein a higher score signifies that the dynamic range of the dehazing result better resembles that of the ground-truth reference. Thus, in image dehazing, high FSIMc and TMQI scores are preferable.

Furthermore, this study employs six public datasets to obtain the quantitative evaluation results, including the FRIDA2 [25], D-HAZY [26], O-HAZE [27], I-HAZE [28], Dense-Haze [29], and 500IMG [14]. The FRIDA2 dataset consists of 330 synthetic images depicting road scenes from the driver’s viewpoint. These images include 66 ground-truth references and 264 hazy images–which are further classified into homogeneous, heterogeneous, cloudy homogeneous, and cloudy heterogeneous according to the haze distribution. Similarly, the D-HAZY dataset is also a synthetic one comprising 1472 indoor ground-truth references. The corresponding 1472 synthetic hazy images are results of applying the simplified Koschmieder model with scene depths captured by a Microsoft’s Kinect camera. In contrast, the remaining four datasets only contain real images. The O-HAZE, I-HAZE, and Dense-Haze consist of 45, 30, and 55 pairs of hazy/haze-free images depicting outdoor, indoor, and both, respectively. Finally, the 500IMG dataset contains 500 natural outdoor images collected from free image-sharing services, such as Google Image, Pinterest, and Flickr.

As discussed earlier in Section 3.2, the haze-free image is not truly free of haze due to the aerial perspective phenomenon. Therefore, in practice, the input image to dehazing systems may be haze-free or hazy with diverse haze conditions. Table 3 then demonstrates the quantitative evaluation results on both hazy and haze-free images to reflect the real-world operating scenario.

It can be observed that the proposed method exhibits the best overall performance. It outperforms the traditional image-processing-based and machine-learning-based methods while demonstrating a relatively significant performance gap with deep-learning-based methods. This superiority is attributed to the excellent performance on haze-free images. In those cases, the piece-wise linear weight often works correctly to modify the dehazing power lest undesirable degradation lowers the image quality. However, even the quantitative evaluation results on hazy images per se demonstrate that the proposed algorithm exhibits a comparative performance to the powerful deep dehazing models. Thus, the quantitative and qualitative evaluation results have proven the efficacy of the proposed algorithm.

## 5. Real-Time Verification

The ultimate objective of image dehazing algorithms is to be integrated into real-world embedded systems, such as self-driving vehicles and intelligent surveillance cameras, which impose strict requirements about power consumption and processing speed. As discussed in Section 1, FPGAs are the best choice for supporting low-power and high-performance computing on embedded systems, and this claim is backed by the work of Wielage et al. [30].

The FPGA verification platform utilized in this study is a Xilinx Zynq-7000 SoC ZC706 Evaluation Kit (Xilinx Asia Pacific Pte. Ltd., Singapore, Singapore). The heart of this platform is an XC7Z045 FFG900-2 SoC device [31], which is partitioned into the processing subsystem (PS) and the programmable logic (PL). The PS comprises a dual-core ARM^®^ Cortex™-A9 processor with a rich set of peripheral interfaces. Meanwhile, the PL mainly consists of configurable logic blocks, on-chip RAMs, and digital signal processing (DSP) slices. It is fabricated with a 28 nm process, and the workhorse executing the proposed algorithm resides therein.

### 5.1. Hardware Implementation

**Figure 11:**
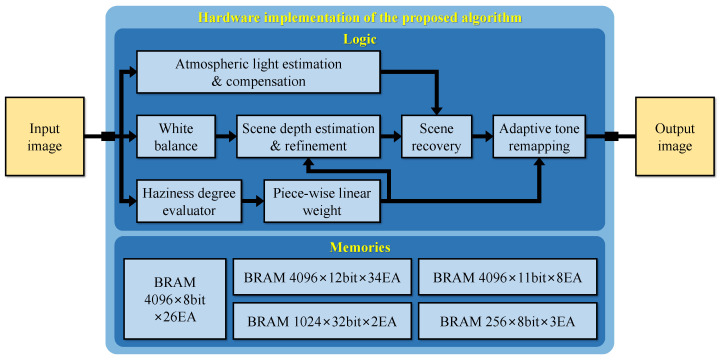
Block diagram of the hardware implementation of the proposed algorithm.

Figure 11 depicts the block diagram of the proposed hardware design. For portability and generality, this study utilizes the Verilog hardware description language (IEEE Standard 1364-2005) [32] to implement the hardware design and Xilinx Vivado Design Suite [33] to verify its performance.

The hardware implementation is partitioned into modules similar to the functional blocks in Figure 2, except that the “scene depth estimation & refinement” module now accounts for extracting saturation and brightness. In modern digital circuit design, it is a standard practice to represent the hardware at the register-transfer level (RTL), a design abstraction that significantly reduces the implementation effort. Additionally, as designing at RTL focuses on modeling the flow of signals between registers, it is not difficult to describe all modules using Verilog.

A good example is the “scene recovery” module that realizes Equation (10) in Section 3.4. This module first utilizes a multiplier to find the product inside the exponential function. This product then serves as the address to access the look-up table (LUT) that realizes the natural exponentiation to get the fraction’s denominator. Meanwhile, three subtractors, corresponding to three color channels, suffice for calculating the numerator. After that, three dividers and three adders complete the “scene recovery” module. Accordingly, an RTL representation of this module is easily attainable using Verilog’s extensive set of arithmetic operators and commands.

Another reason for this study to use Verilog is to ease the integration of existing designs into the proposed hardware implementation. Ngo et al. [14] and Cho et al. [21] provided the Verilog implementation of two modules: “atmospheric light estimation & compensation” and “adaptive tone remapping” in the forms of deliverable intellectual properties (IPs). Therefore, these two IPs can be easily utilized in a “plug-and-play” manner. Concerning memory usage, the “atmospheric light estimation & compensation” IP employs three 256×8-bit memories to store the RGB values of the corresponding 256 atmospheric light candidates. Meanwhile, the “adaptive tone remapping” IP utilizes two 1024×32-bit memories to construct the cumulative distribution function of the luminance to calculate the luminance gain $G_{L}$ in Equation (11).

**Figure 12:**
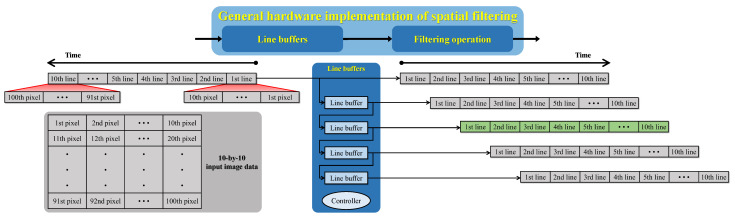
Block diagram of the general hardware implementation of the spatial filtering operation. A 10-by-10 input image data and a 5-by-5 filtering kernel were assumed.

Back to the example of “scene recovery,” because this module only involves simple arithmetic operations, its implementation does not require memory accesses. In other modules, such as the “scene depth estimation & refinement,” data exchange between digital circuits and memories is essential to realize the spatial filtering operation. More precisely, this study employs the modified hybrid median filter (mHMF) to refine the scene depth estimate because the mHMF possesses an excellent edge-preserving smoothing characteristic. Despite such an important role in the proposed algorithm, it often requires a considerable effort to design a fast and efficient image filter in hardware. The underlying difficulty is to handle the image data stream to obtain all necessary pixels covered by the filtering kernel. In a previous work by Park and Kim [34], they leveraged a cascaded arrangement of flip-flops to address that difficulty. Unfortunately, this approach is not a general solution because it cannot handle variable-sized images. Therefore, this study presents a general hardware architecture for implementing any spatial filtering operations, as Figure 12 illustrates.

The hardware implementation of a spatial filter can be decomposed into two main modules: “line buffers” and “filtering operation.” The former consists of line buffers whose depth is equal to the image’s width so that each of them can delay the image data stream by one line. The reason for using line buffers is to remedy the inherent problem of cascaded flip-flops, that is, the inability to handle variable-sized images. However, it is worth clarifying that the proposed hardware implementation can process variable-sized images of the maximum 4K resolution. Accordingly, the depth of line buffers is set to 4096, accounting for a large number of 4096×8-bit, 4096×12-bit, and 4096×11-bit memories in Figure 11. Figure 12 demonstrates the operation of the “line buffers” module when considering a 10-by-10 image and a 5-by-5 filtering kernel. Consequently, this implementation requires four line buffers, and the output of the second (displayed in green) is the reference line indicating the availability of filtering results. Furthermore, unlike flip-flops, line buffers require timing signals for reading/writing operations. Therefore, beneath four line buffers lies the controller responsible for this task.

Compared to the “line buffers” module, the “filtering operation” is application-dependent and relatively simple. For example, multipliers and adders are adequate for implementing the typical moving average filter. Back to mHMF in the “scene depth estimation & refinement” module, this study utilizes the optimized merging sorting network (proposed by Ngo et al. [14]) to realize its “filtering operation.”

### 5.2. Implementation Results

Given the hardware implementation described above, this study utilizes Xilinx Vivado Design Suite [33] to map it onto an XC7Z045 FFG900-2 SoC device [31]. The implementation results are shown in Table 4, from which it can be observed that the proposed design consumes 15.88%, 30.39%, and 16.33% of registers, LUTs, and memories, respectively. This hardware resource utilization signifies that it fit compactly into the target FPGA device. Meanwhile, the remaining space is sufficient for implementing other high-level image processing algorithms, such as object recognition.

In addition, the considerable number of registers utilized for implementation is to achieve a maximum operating frequency of 273.90 MHz. Notably, Xilinx Vivado v2019.1 does not provide this piece of information directly. Instead, it is calculated based on the target clock period *T* and the worst negative slack WNS, as shown in Equation (13). Substituting the maximum operating frequency $f_{max}$ into Equation (14) yields the maximum processing speed MPS in terms of fps. In the denominator, {H,W} denotes the height and width of images, and $\left\{B_{H},B_{V}\right\}$ the horizontal and vertical blank periods, respectively. As the proposed design can function properly with $\left\{B_{H},B_{V}\right\}$ of at least one clock cycle and one image line, all necessary information for calculating the maximum processing speed is now available. For example, the DCI 4K resolution requires 8,853,617 clock cycles (=4097×2161) to handle a whole frame; hence, the proposed design can achieve 30.94 fps ($\approx 273.90\times 10^{6}/8,853,617$), as shown in Table 5. This maximum speed is adequate for real-time processing of videos encoded by Phase Alternation by Line and National Television System Committee standards [35].

(13) $$\begin{array}{ccc} f_{max} & = & \frac{1}{T-WNS}, \end{array}$$

(14) $$\begin{array}{ccc} MPS & = & \frac{f_{max}}{\left(H+B_{V}\right)\left(W+B_{H}\right)}. \end{array}$$

Table 6 demonstrates implementation results of the proposed design alongside those of two existing ones, developed by Park and Kim [34] and Ngo et al. [14]. The design by Park and Kim [34] is the implementation of the DCP with a fast estimation of atmospheric light, while the design by Ngo et al. [14] directly implements the ICAP. It can be observed that the proposed design is faster but consumes more hardware resources than these two benchmark implementations. However, this increase in hardware resource consumption renders the proposed dehazing system robust against various haze conditions; hence, bringing about superior performance, as demonstrated in Section 4. Additionally, although the design by Park and Kim [34] attains the maximum operating frequency of 88.70 MHz, it can only handle videos of super VGA resolution (800×600) because it utilizes cascaded flip-flops to realize spatial filtering operations. In contrast, the remaining two exploit the general implementation so that they can process variable-sized images of up to DCI 4K resolution.

**Figure 13:**
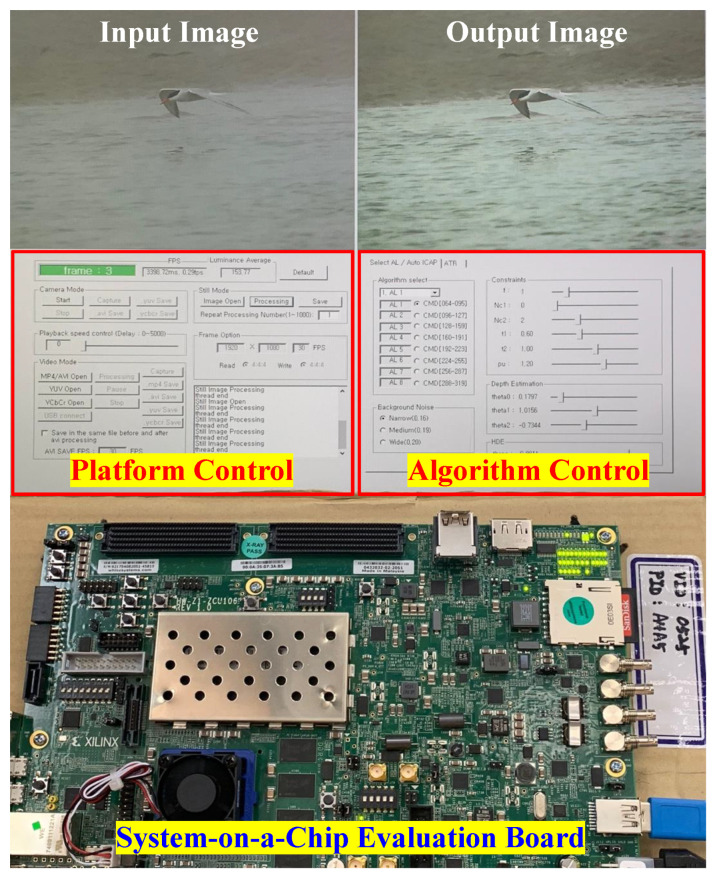
Real-world execution of the proposed dehazing system.

To complete this section, Figure 13 demonstrates the real-world execution of the proposed dehazing system. The upper half of this figure is the C platform undertaking the communication between the host computer and a Xilinx Zynq-7000 SoC ZC706 Evaluation Kit (Xilinx Asia Pacific Pte. Ltd., Singapore, Singapore). The C platform consists of three main panels for ease of interaction. The first occupies the upper half and displays the input-output data side-by-side for qualitative assessment, while the second is the platform control located in the bottom-left. This panel allows users to select the data source from still images, videos, and live cameras. It also supports fundamental operations that are common in video players, such as pause, stop, speed control, and save. The last is the algorithm control that comprises slide bars and buttons to configure the hardware implementation. Hence, the C platform provides a convenient means of verifying the real-time operation of the proposed dehazing system.

## 6. Conclusions

This paper presented a robust image dehazing algorithm that could deliver satisfactory performance in various haze conditions. This robustness is a result of adopting the haze density estimate to modify the scene depth estimator. More specifically, this study discriminated the haze condition of arbitrary input images based on their haze density estimate. After that, the piece-wise linearization method yielded a corresponding weight factor to modify the scene depth estimator based on the discrimination result. As haze strongly correlates with the scene depth, the previous action implicitly modifies the dehazing power. Consequently, the proposed algorithm could handle images with different haze conditions, such as haze-free, mildly, moderately, and densely hazy. An extensive evaluation against six benchmark methods demonstrated the superiority of the proposed algorithm.

Moreover, this paper also presented a real-time hardware implementation targeted on a Xilinx XC7Z045 FFG900-2 SoC device. This implementation could handle variable-sized images with the maximum DCI 4K resolution, attributed to using line buffers instead of cascaded flip-flops. This study then utilized Xilinx Vivado Design Suite to synthesize and map the hardware implementation onto the target FPGA device. The implementation results demonstrated that the proposed design could attain the maximum processing speed of 30.94 fps for DCI 4K resolution; hence, showing a great potential for integration into high-performance, high-quality image processing systems.

## Figures and Tables

**Table 1:** Summary of benchmark methods for performance assessment.

Proposed by	Core Technique	Category	Abbreviation
Tarel and Hautiere [22]	Spatial filtering	Traditional image processing	FDH
He et al. [10]			DCP
Zhu et al. [7]	Maximum likelihood estimates	Machine learning	CAP
Ngo et al. [14]			ICAP
Cai et al. [17]	Convolutional neural network	Deep learning	DNet
Ren et al. [18]			MNet

**Table 2:** Parameter configuration of the proposed algorithm.

Parameter	Description	Value
$\left\{\theta_{0},\theta_{1},\theta_{2}\right\}$	Coefficients of the depth estimator	$\left\{0.1800,1.0147,-0.7350\right\}$
$\left\{\Gamma_{1},\Gamma_{2}\right\}$	Thresholds for constructing the piece-wise linear weight	$\left\{0.8811,0.9344\right\}$
$\omega_{A}$	Compensation scaling factor	0.6000

**Table 3:** Average quantitative results on different datasets. Top three results are boldfaced in red, green, and blue. P.A. stands for the proposed algorithm.

	Method	FSIMc							TMQI						
Dataset		FDH	DCP	CAP	ICAP	DNet	MNet	P.A.	FDH	DCP	CAP	ICAP	DNet	MNet	P.A.
FRIDA2	Hazy	0.7807	0.7746	0.7918	0.7984	0.7963	0.8009	0.7975	0.7314	0.7291	0.7385	0.7400	0.7336	0.7232	0.7326
	Haze-free	0.8566	0.9586	0.9102	0.9382	0.9703	0.9656	0.9916	0.9329	0.9680	0.8832	0.9747	0.8716	0.9024	0.9184
D-HAZY	Hazy	0.8703	0.9002	0.8880	0.8787	0.8874	0.8822	0.8811	0.8000	0.8631	0.8206	0.8165	0.7966	0.8023	0.7976
	Haze-free	0.8672	0.9541	0.8968	0.9402	0.9843	0.9497	0.9904	0.8877	0.9123	0.8829	0.9255	0.9073	0.9075	0.9125
O-HAZE	Hazy	0.7733	0.8423	0.7738	0.8219	0.7865	0.8553	0.8280	0.8416	0.8403	0.8118	0.8935	0.8413	0.8737	0.8891
	Haze-free	0.8379	0.9645	0.8679	0.8930	0.9839	0.9369	1.0000	0.8172	0.8765	0.7906	0.8470	0.8562	0.8513	0.9324
I-HAZE	Hazy	0.8055	0.8208	0.8252	0.8482	0.8482	0.8631	0.8675	0.7740	0.7319	0.7512	0.7892	0.7598	0.7819	0.8147
	Haze-free	0.8283	0.9335	0.8716	0.9277	0.9751	0.9724	0.9997	0.8380	0.8106	0.7681	0.8979	0.8343	0.8543	0.8960
Dense-Haze	Hazy	0.5598	0.6419	0.5773	0.5703	0.5573	0.6029	0.5864	0.5627	0.6383	0.5995	0.5824	0.5723	0.6176	0.5985
	Haze-free	0.8571	0.9414	0.8508	0.9131	0.9776	0.9693	0.9985	0.8440	0.8611	0.7742	0.8790	0.8539	0.8632	0.9190
500IMG	Haze-free	0.8645	0.9563	0.8795	0.9218	0.9870	0.9383	0.9992	0.8138	0.8858	0.8438	0.8685	0.8775	0.8605	0.8970
Total	Hazy	0.7573	0.7746	0.7693	**0.7761**	0.7725	**0.7896**	**0.7826**	0.7294	**0.7357**	0.7336	**0.7352**	0.7312	0.7341	**0.7413**
	Haze-free	0.8621	**0.9548**	0.8798	0.9206	**0.9840**	0.9449	**0.9982**	0.8293	**0.8802**	0.8297	**0.8730**	0.8730	0.8652	**0.9033**
	Overall	0.8170	**0.8886**	0.8392	0.8675	**0.9063**	0.8879	**0.9191**	0.7863	**0.8272**	0.7944	**0.8224**	0.8209	0.8171	**0.8438**

**Table 4:** Hardware implementation results of the proposed image dehazing algorithm. The symbol # denotes quantities.

Xilinx Vivado v2019.1			
Device	XC7Z045-2FFG900-2		
Slice Logic Utilization	Available	Used	Utilization
Slice registers (#)	437,200	69,440	15.88%
Slice LUTs (#)	218,600	66,442	30.39%
RAM36E1/FIFO36E1s	545	89	16.33%
Minimum period	3.65 ns		
Maximum frequency	273.90 MHz		

**Table 5:** Maximum processing speed for different video resolutions. The symbol # represents quantities.

Video Resolution		Frame Size	Required Clock Cycles (#)	Processing Speed (fps)
Full HD (FHD)		1920×1080	2,076,601	131.90
Quad HD (QHD)		2560×1440	3,690,401	74.22
4K	UW4K	3840×1600	6,149,441	44.54
	UHD TV	3840×2160	8,300,401	33.00
	DCI 4K	4096×2160	8,853,617	30.94

**Table 6:** Comparison with existing hardware designs. The symbol # represents quantities.

Hardware Utilization	Park and Kim [34]	Ngo et al. [14]	Proposed Design
Registers (#)	53,400	57,848	69,440
LUTs (#)	64,000	53,569	66,442
DSPs (#)	42	0	0
Memory (Mbits)	3.2	2.4	2.8
Maximum operating frequency (MHz)	88.70	271.67	273.90

## Data Availability

Data available in a publicly accessible repository. The data presented in this study are openly available in [25,26,27,28,29] and FigShare at https://doi.org/10.6084/m9.figshare.14729052.v1, accessed on 18 August 2021.

## Appendix A

This appendix provides details of the calculation of the haze concentration estimate (denoted as $\Gamma_{I}$ in Section 3.2). Firstly, Ngo et al. [8] formulated a transmittance-dependent optimization problem based on four haze-relevant features (dark channel, saturation, brightness, and sharpness), denoted as O(t) in Equation (A1). The sharpness $\sigma_{I}$ therein can be easily obtained as the standard deviation of the input image I. Furthermore, for ease of manipulation, Ngo et al. [8] assumed that the atmospheric light possessed equal intensities across color channels; hence, the plain representation *A*. In this study, *A* can be set to the average of the estimate obtained in Section 3.3. Finally, Ngo et al. [8] adopted a regularization term, κ/t, with κ being a regularization parameter, to ensure that the optimization is analytically solvable.

(A1) $$\begin{array}{ccc} O[t(x)] & = & \frac{I_{mc}\left(x\right)\sigma_{I}\left(x\right)}{t\left(x\right)[At\left(x\right)-A+I_{m\Omega }\left(x\right)]}+\frac{\kappa }{t(x)}, \end{array}$$

(A2) $$\begin{array}{ccc} I_{mc}\left(x\right) & = & \max_{c\in \{R,G,B\}}I^{c}\left(x\right)-\min_{c\in \{R,G,B\}}I^{c}\left(x\right), \end{array}$$

(A3) $$\begin{array}{ccc} I_{m\Omega }\left(x\right) & = & \min_{y\in \Omega (x)}\left[\min_{c\in \{R,G,B\}}I^{c}\left(y\right)\right]. \end{array}$$

Given the information above, it is possible to find the closed-form expression of the transmittance $\hat{t}$ that maximizes O(t). The haze concentration estimate $\Gamma_{I}$ is then derived from $\hat{t}$ as follows:

(A4) $$\begin{array}{ccc} \Gamma_{I} & = & \frac{1}{\left|\Psi \right|}\sum_{\forall x\in \Psi }\left[1-\hat{t}\left(x\right)\right]\\ & = & \frac{1}{\left|\Psi \right|}\sum_{\forall x\in \Psi }\left\{\frac{1}{A}\left[I_{m\Omega }+\frac{I_{mc}\sigma_{I}}{\kappa }-\sqrt{\frac{I_{mc}\sigma_{I}}{\kappa }\left(\frac{I_{mc}\sigma_{I}}{\kappa }-A+I_{m\Omega }\right)}\right]\right\}, \end{array}$$

where Ψ denotes the entire image domain, as mentioned earlier in Section 3.2.

## Appendix B

This appendix summarizes the calculation of $G_{L}$, $G_{C}$, $W_{L}$, and $W_{C}$ in Equations (11) and (12). For ease of explanation, it is convenient to define an auxiliary function $f_{\mathrm{CDF}}\left(L,\varphi \right):R^{H\times W}\to R$ that takes two input data (the luminance *L* of size H×W and a scalar 0≤φ≤1), computes the cumulative distribution function (CDF), and returns the luminance intensity where the CDF value is equal to φ.

Cho et al. [21] defined the luminance gain $G_{L}$ as follows:

(A5) $$G_{L}=\frac{L}{2^{11}}{\left[255{\left(1-\frac{L-\mathrm{ALP}}{255}\right)}^{\phi }\left(\frac{255-L}{255}\right)\right]}^{2},$$

where ALP and ϕ are the adaptive limit point and the adaptive exponent, calculated using Equations (A6) and (A7), respectively. The representation $\bar{L}$ utilized in these two denotes the average of *L* over the entire image domain.

(A6) $$\mathrm{ALP}=\{\begin{array}{cc} 0.04+\frac{0.02}{255}\left[f_{\mathrm{CDF}}\left(L,0.9\right)-f_{\mathrm{CDF}}\left(L,0.1\right)\right] & \bar{L}>0.5\\ 0.04-\frac{0.02}{255}\left[f_{\mathrm{CDF}}\left(L,0.9\right)-f_{\mathrm{CDF}}\left(L,0.1\right)\right] & \mathrm{otherwise} \end{array}$$

(A7) $$\phi =\frac{1.5}{\bar{L}-f_{\mathrm{CDF}}\left(L,0.1\right)}$$

Next, Cho et al. [21] defined the adaptive luminance weight $W_{L}$ as a linear function of *L*, $W_{L}=a_{L}L+b_{L}$, where $a_{L}$ and $b_{L}$ are user-defined parameters representing the slope and intersection.

Meanwhile, the chrominance gain $G_{C}$ is formulated as $G_{C}=\left(L_{e}/L\right)\cdot C$ to emphasize the color in proportion to the luminance enhancement, as introduced in Section 3.4. Similarly, the adaptive chrominance weight $W_{C}$ is also defined based on the luminance information as follows:

(A8) $$W_{C}=\{\begin{array}{cc} 0.7 & L<{\mathrm{TH}}_{1}\\ 0.7-0.26\;\left(\frac{L-{\mathrm{TH}}_{1}}{{\mathrm{TH}}_{2}-{\mathrm{TH}}_{1}}\right) & {\mathrm{TH}}_{1}\leq L\leq {\mathrm{TH}}_{2}\\ 0.44 & \mathrm{otherwise} \end{array}.$$
